# International Clinics

**Published:** 1891-11

**Authors:** 


INTERNATIONAL CLINICS. A Quarterly of Clinical Lectures by Professors and Lecturers in the Leading Medical Colleges of the U. S., Eng. and Canada: J. B. Lippincott Co., Phila. 1891. Vol. 2.

With a memoir of the late Dr. Joseph Leidy of Philadelphia.

The second volume of this highly interesting and valuable series of clinical lectures contains many very important subjects. We note particularly one by Dr. Lawson of London, on the pathology of Angina Pectoris, which will be found very instructive.

Prof. Mays of Philadelphia, contributes a very interesting discussion on Asthma, which will prove highly instructive to many.

An important lecture on "Electricity in Neurasthenia and other Functional Neuroses" is given, which will be invaluable to those wishing to keep posted in regard to the growing importance of electricity in therapeutics and surgical procedures.

Altogether this volume is well entitled to the fullest attention. R. W. W.

